# Development and aetiology of body dissatisfaction in adolescent boys and girls

**DOI:** 10.1080/02673843.2014.985320

**Published:** 2014-12-03

**Authors:** Jacinthe Dion, Marie-Eve Blackburn, Julie Auclair, Luc Laberge, Suzanne Veillette, Marco Gaudreault, Patrick Vachon, Michel Perron, Évelyne Touchette

**Affiliations:** ^a^ Département des Sciences de la Santé, Université du Québec à Chicoutimi, Chicoutimi, Québec, Canada; ^b^ ÉCOBES Recherche et Transfert, Cégep de Jonquière, Saguenay, Québec, Canada; ^c^ Département des Sciences Humaines, Université du Québec à Chicoutimi, Saguenay, Québec, Canada; ^d^ Département de psychoéducation, Université du Québec à Trois-Rivières, Trois-Rivières, Québec, Canada

**Keywords:** adolescent, body image, body dissatisfaction, body mass index, diet, weight loss

## Abstract

This longitudinal study aims to describe the development of body dissatisfaction (BD), measured with the *Contour Drawing Rating Scale,* between the ages of 14 and 18, and to identify factors associated with BD at age 18, among 413 adolescents. Between the ages of 14 and 18, the proportion of girls wanting to be thinner increased, although it remained unchanged among boys. A ratio of 1:2 girls and 1:5 boys reported having seriously tried to lose weight. Factors associated with BD in girls at age 18 were (1) wanting to be thinner, (2) body mass index (BMI), (3) weight control behaviours and (4) negative comments about weight. Factors associated with BD in boys at age 18 were (1) wanting to be thinner or bigger, (2) BMI, (3) having experienced sexual intercourse and (4) negative comments about weight. The high prevalence of BD and weight-related concerns suggest a need for early interventions.

## Introduction

In this study, body dissatisfaction (BD) is the discrepancy between the perceived shape and the ideal one. BD is a serious public mental health problem; its prevalence ranges from 57% to 84% among adolescent girls and from 49% to 82% among adolescent boys across many studies (Almeida, Severo, Araújo, Lopes, & Ramos, 2012; Chen, Fox, Haase, & Ku, 2010; Cortese et al., 2010; Lawler & Nixon, 2011; Petroski, Pelegrini, & Glaner, 2012). Given its high prevalence, factors associated with its development should be further studied.

BD is part of a larger concept of body image, which is viewed as a multidimensional construct (Thompson, Heinberg, Altabe, &Tantleff-Dunn, 1999; Wertheim & Paxton, 2011). According to Menzel, Krawczyk, and Thompson (2011), attitudinal body image can be classified into four components: global subjective satisfaction or disturbance, affective distress regarding one's appearance, cognitive aspects of body image and behavioural ones. Body image can thus refer to image satisfaction–dissatisfaction, feelings about one's appearance, concerns about various body parts and physical characteristics, etc. Dissatisfaction related to body image is an important risk factor for developing psychological problems, such as depression, suicidal ideation, low self-esteem, unhealthy weight control behaviours as well as eating pathologies (Crow, Eisenberg, Story, & Neumark-Sztainer, 2008; Neumark-Sztainer, Paxton, Hannan, Haines, & Story, 2006b; Paxton, Neumark-Sztainer, Hannan, & Eisenberg, 2006b; Stice, 2002; Stice & Bearman, 2001; Stice, Marti, & Durant, 2011; Tiggemann, 2005). Moreover, a meta-analysis suggested that it may be the most consistent and robust risk and maintenance factor for eating pathologies (Stice, 2002).

This study focuses on BD evaluated based on the discrepancy between the perceived shape and the ideal one, using silhouette images depicting different shapes, weights and sizes of the human body, ranging from very thin to excessively overweight. There are several advantages to figural drawing scales, including ease and flexibility in administration (Gardner & Brown, 2010). These scales enable us to assess both the desire to be thinner and the desire to be bigger for both genders. Several studies have been conducted over the past decades on body image. However, there is a paucity of longitudinal studies exploring BD with a figure rating scale.

### Differences in BD between girls and boys

Notwithstanding the existence of BD among both genders, boys are underrepresented in published literature on BD. Among the longitudinal studies investigating BD in both genders (which did not use silhouette figures in varying shapes in their assessment), most have found that BD increases with age among girls and boys alike (e.g. Bucchianeri, Arikian, Hannan, Eisenberg, & Neumark-Sztainer, 2013; Calzo et al., 2012; Mendelson, White, & Mendelson, 1996), although one study found it to decrease in boys (Bearman, Presnell, Martinez, & Stice, 2006). It is also well documented in the literature that girls report greater BD than boys (e.g. Eisenberg, Neumark-Sztainer, & Paxton, 2006). In her longitudinal study on BD in girls, Tiggemann (2005) found that girls perceive themselves as progressively more overweight and dissatisfied with their shape throughout their adolescence.

### Factors associated with BD

BD has been conceptualised within a bio-psycho-social multidimensional framework (Cash & Pruzinsky, 2011) that includes psychological, physical and environmental factors. Over the past decades, several cross-sectional and longitudinal studies have found various factors associated with body image dissatisfaction. However, only a few longitudinal studies have included physical, psychological as well as social risk factors of BD (measured with silhouette figures) during adolescence. One prospective study with adolescent girls showed that even if reciprocal friends were initially similar on BD and dieting constructs, BD levels of the initial friendship group did not predict individual BD and dieting variables 1 year later (Woelders, Larsen, Scholte, Cillessen, & Engels, 2010). Cross-sectional studies indicate that body mass index (BMI) is associated with BD, especially in girls (Cortese et al., 2010; Jones, Fries, & Danish 2007). In the Cortese et al. (2010) study, BD was found not only in overweight and underweight male and female adolescents, but also among average-weight and even slightly underweight girls. Internalisation of appearance ideals, peer appearance criticisms and conversations with friends about appearance were also found to be related to BD (Lawler & Nixon, 2011). In a Taiwanese study (Chen et al., 2010), BMIs, perceived physical appearance and internalisation of socio-cultural ideals, were associated with BD.

Results from longitudinal studies focusing on BD using measures other than a figure rating scale revealed mixed findings on BD risk factors. Concerning physical factors, BMI was found to be one of the strongest and most consistent predictors of BD among adolescents in all recent studies (Lawler & Nixon, 2011; Paxton, Eisenberg, & Neumark-Sztainer, 2006a; Wilkosz, Chen, Kenndey, & Rankin, 2011) but one (Bearman et al., 2006). Self-reported dietary restraint also predicted BD (Bearman et al., 2006). Some longitudinal studies have found several psychological risk factors of BD, such as low self-esteem (Cantin & Stan, 2010; Paxton et al., 2006a), depression (Paxton et al., 2006a) and negative affectivity (Bearman et al., 2006; Presnell, Bearman, & Stice, 2004). Nonetheless, some studies did not find depression (Holsen, Kraft, & Roysamb, 2001; Stice & Whitenton, 2002) or low self-esteem (Tiggeman, 2005) to prospectively predict BD. Finally, some social and family-related factors are reported to predict BD in longitudinal studies, for example, friends dieting and weight teasing (Paxton et al., 2006a), and family connectedness, but only among girls (Crespo, Kielpikowski, Jose, & Pryor, 2010).

Although many studies have been conducted on body image among girls over the past decades, only a few longitudinal ones have examined factors associated with BD and its development during adolescence among both girls and boys using a figure rating scale. Yet, this is a critical period for body image construction. This study uses longitudinal data spanning ages 14–18 years (which is a longer period than most previously cited studies) to investigate the development of BD over time, as well as to identify associated factors using a bio-psycho-social framework. To compare girl's and boy's experience with BD, adolescent boys and girls were both included in the sample. This study, which was conducted in Canada, adds to the previous data collected mainly in the USA and Australia. The objectives of this study were (1) to describe the development of BD at ages 14, 16 and 18, and (2) to identify factors associated with BD at age 18 for girls and boys. As suggested by Presnell et al. (2004), BD was assessed in both directions: the desire to be thinner and the desire to be bigger. This allowed us to evaluate the factors involved in both aspects of BD. A better understanding of BD during adolescence would be useful for early interventions promoting the development of a healthy body and self-image throughout adolescence.

## Materials and methods

### Participants

The study sample included 605 students (55.4% girls and 44.6% boys) aged 14 years (2002), attending public and private high schools in the Saguenay–Lac-Saint-Jean region (Northern Québec, Canada). These students were interested in participating in the longitudinal study and were authorised to do so by their parents. The study was approved by the Public Health Ethics Committee of the Quebec Ministry of Health and Social Services. Informed consent was obtained from parents and adolescents. Among the adolescents, 68.3% (*n* = 413, 63.2% girls) participated in the data collection at age 18 in 2006, at school or by mail. Overall, 413 adolescents constituted the sample.

Statistical analyses were carried out to compare the 413 participants with the 192 adolescents from the initial sample who did not participate at age 18. Overall, more girls than boys participated (*p* < 0.000) in the longitudinal study. No statistical difference was observed between the two groups in terms of socio-economic condition (*p* = 0.61) or parental education (*p* = 0.17 for mothers; *p* = 0.86 for fathers). No difference was found between participants and non-participants in terms of baseline levels of self-esteem and negative comments. However, participants were more likely to have a higher BD baseline (*p* < 0.000) and to have tried to lose or control weight (*p* = 0.001).

### Measures

#### Body dissatisfaction

A figure rating scale was used to measure BD. Participants were required to select one of nine different images of female and male bodies ranging from thin to overweight to depict (1) their perceived body shape and (2) their ideal body shape (*Contour Drawing Rating Scale*; Thompson & Gray, 1995). BD was derived from this measure, i.e. when perceived and ideal shapes differed. The degree of discrepancy varied between − 8 and − 1 for those wanting a thinner shape and between 1 and 8 for those wanting a bigger shape. A score of 0 was interpreted as body image satisfaction. This scale had good test–retest reliability among 32 female university students (Thompson & Gray, 1995), and also among 1056 early adolescent girls (Wertheim, Paxton, & Tilgner, 2004). Validity was also demonstrated among female adults (Thompson & Gray, 1995), female and male adults (Thompson, 1993), and early adolescent girls (Wertheim et al., 2004). In our sample, BMI was found to be highly correlated with perceived shape among girls (*r* = 0.70, *p* < 0.000) and boys (*r* = 0.71, *p* < 0.000), indicating consistency between participants’ perceived shape and self-reported BMI. A three-item measure of general body image dissatisfaction (dissatisfaction with physical appearance, body and clothing) was also included to complement the figure rating scale (which measures BD). A high correlation was found between general body satisfaction and BD at T1 (*r* = 0.49, *p* < 0.000) and T2 (*r* = 0.47, *p* < 0.000).

#### Potential factors associated with BD

The French version of Rosenberg's 10-item Self-Esteem Scale (Rosenberg, 1965; Vallières & Vallerand, 1990) was used to assess youth's self-esteem at 14 and 18 years. Items are scored on a four-point Likert scale (1 = ‘strongly disagree’ to 4 = ‘strongly agree’). The total score varies from 10 to 40, with a high score reflecting a high level of self-esteem. Internal consistencies range from 0.70 to 0.88 in French-Canadian populations (Vallières & Vallerand, 1990). Cronbach's α in our sample was 0.85 at T1 and 0.89 at T2.

At 14 and 18 years of age, adolescents were asked whether they had seriously tried to lose or control their weight in the past 6 months (0 = no and 1 = yes). At 14 years of age, they were also asked which weight control strategies they had tried (i.e. skipping meals, using laxatives) and whether they had received negative comments about their weight from their mother, father, sibling, friends, physical education teacher or others. A recoding was performed to compute the number of persons who had made negative comments (0 = none, 1 = 1 person, 2 = 2 persons or more). At 18 years of age, they were asked whether they had experienced sexual intercourse (0 = no and 1 = yes).

Finally, at 18 years of age, self-reported height and weight were used to compile their BMI. The BMI was calculated as weight (kg) divided by the square of the height (m^2^). The score was categorised to define weight status according to the body weight classification system for Canadian adults, based on the World Health Organization's system (Health Canada, 2003).

### Statistical analyses

All analyses were conducted separately for boys and girls, as previous research suggested differences in risk factors across genders. Descriptive analyses were conducted using chi-square tests (for dichotomous variables) and Spearman correlations (for ordinal and continuous variables). Because of the type of data and their distribution, as well as to increase robustness (Tomkins, 2006), Cochran's non-parametric test (for dichotomous variables) and Friedman's non-parametric test (for ordinal and continuous variables) were used to test the effect of time. BD was defined as either (1) a desire to be thinner or (2) a desire to be bigger. Multinomial logistic regressions were performed for the boys, to identify factors at age 14 that predicted BD (i.e. wanting a thinner or a bigger shape) at age 18, and factors at age 18 associated with BD at age 18. Among the girls, only 12 wanted a bigger shape. Consequently, statistical analyses (logistic regressions) were limited to those wanting a thinner shape.

## Results

### Development of BD between ages 14 and 18

Figure 1(a) shows the distribution of adolescent girls’ perceived shape within each age group as well as the distribution of BD. Overall, the number of girls who perceived themselves as bigger increased from ages 14 to 18 [χ^2^(1) = 19.69, *p* < 0.000]. The proportion of adolescent girls dissatisfied with their body image remained (*p* = 0.73) between ages 14 and 18. More specifically, 57.1% of girls wanted a thinner shape at age 14, and the proportion increased to 64.8% at 18 years [*Q*(1) = 8.05, *p* < 0.01]. Conversely, the proportion of girls wanting a bigger shape decreased from 13.4% to 4.6% for the same period [*Q*(1) = 18.24, *p* < 0.000].Figure 1 (a) Proportions of perceived shape and BD among girls. (b) Proportions of declared shape and BD among boys.

**Figure:**
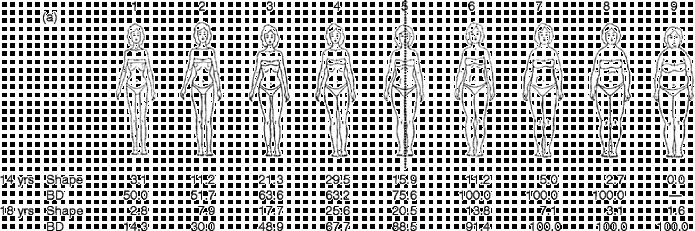

**Figure:**
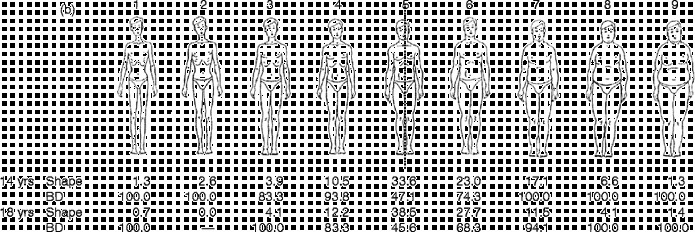


From ages 14 to 18, the distribution of perceived shapes did not change in adolescent boys (*p* = 0.49) (see Figure 1(b)). The proportion of those dissatisfied with their body image remained (*p* = 0.17) between ages 14 and 18. More specifically, the desire for a thinner shape (44.0% at 14 years to 38.5% at 18 years; *p* = 0.17) and the desire to be bigger remained almost unchanged (30.7% at 14 years to 29.1% at 18 years; *p* = 0.72).

### Associations between BD and BMI

A strong association was found between BD and BMI for both adolescent girls (χ^2^ = 54.98, *p* < 0.000) and boys (χ^2^ = 52.73, *p* < 0.000) at 18 years. As expected, almost all overweight girls (96.4%) wanted a thinner shape (see Figure 2(a)). Interestingly, 68.0% of normal-weight girls and 18.8% of underweight girls also wanted a thinner shape. Among boys, the situation was different. A proportion of 36.3% of normal-weight boys and 71.4% of underweight boys wanted a bigger shape (see Figure 2(b)). The profile of overweight boys was similar to that of overweight girls in that more of them wanted a thinner shape.Figure 2 (a) BD according to BMI at 18 years among girls. (b) BD according to BMI at 18 years among boys.

**Figure:**
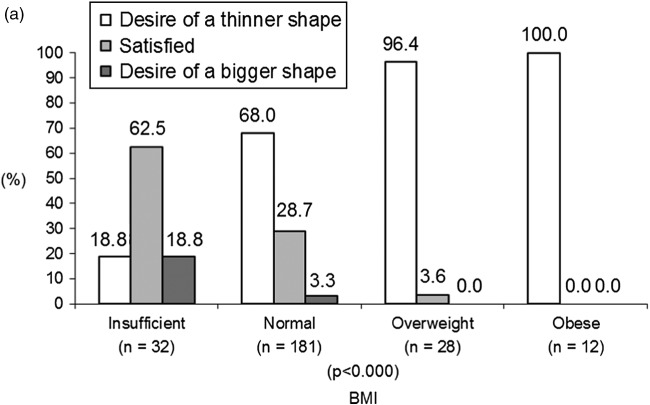

**Figure:**
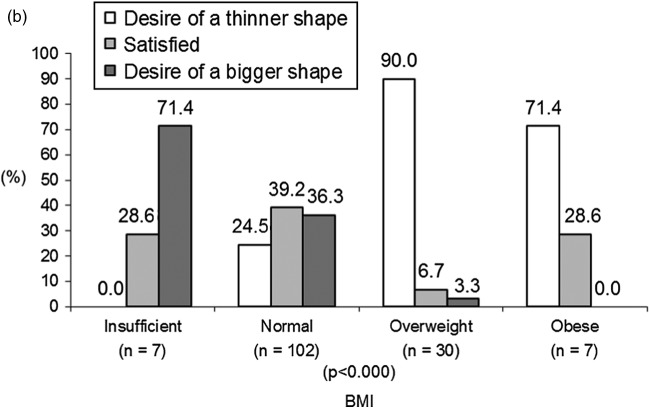


### Strategies used to lose weight

Approximately one of two girls tried to lose or control her weight (48.4% at 14 and 51.9% at 18; *p* = 0.44), while fewer boys tried to do so (20.7% at 14 and 22.0% at 18; *p* = 0.86). At 14 years, adolescents were asked which strategies they used to lose or control their weight. Among the 48.4% of girls (*n* = 122) who tried to lose or control their weight at 14 years, the majority did so by reducing sugar and fat intake (94.2%). Also, the majority engaged in intense exercise (78.5%) or skipped meals (53.7%). More excessive weight control behaviours were also adopted, such as fasting (32.2 %), starting or resuming smoking (19.8%), using appetite suppressants (10.0 %), purging after a meal (9.9%) and using laxatives (6.7%). Only 20.7% of boys (*N* = 30) tried to lose or control their weight at 14 years of age, by reducing sugar and fat intake (79.3%) and engaging in intense exercise (83.3%). Excessive weight control behaviours were rarely or never adopted (e.g. using appetite suppressants or laxatives, starting or resuming smoking, purging after a meal).

### Factors associated with wanting a thinner shape in girls at age 18

Logistic regression analyses were conducted to predict the desire for a thinner shape at age 18. Three risk factors at age 14 predicted the desire for a thinner shape at age 18 among girls (see Table 1). The likelihood of wanting a thinner shape at 18 years was almost 3.8 times higher for girls wanting a thinner shape at 14 years (odds ratio (OR) = 3.78, confidence interval (CI) = 1.75–8.17, *p* < 0.01), 2.2 times higher for girls who seriously tried to lose or control weight at age 14 (OR = 2.18, CI = 1.11–4.28, *p* < 0.05) and 1.7 times higher for girls who received negative comments about their weight at age 14 (OR = 1.73, CI = 1.01–2.95, *p* < 0.05) compared with girls who did not report these behaviours. Finally, self-esteem was not statistically significant.

**Table 1:** Factors measured at 14 years old predicting BD at 18 years old among girls.

	Desire to be thinner at 18		
Variable	SE	OR	95% CI
BD			
Desire to be thinner at 14	0.39	3.78^*^^*^	1.75–8.17
Desire to be bigger at 14	0.51	1.56	0.57–4.23
Attempt to lose or control their weight	0.35	2.18^*^	1.11–4.28
Receiving negative comments about their weight	0.27	1.73^*^	1.01–2.95
Self-esteem	0.04	1.07	1.00–1.15

Notes: *N* = 232. ^*^ *p* < 0.05; ^*^^*^ *p* < 0.01.

Two risk factors at age 18 were found to be associated with the desire for a thinner shape at age 18: weight control behaviours and BMI (see Table 2). The likelihood of wanting a thinner shape at 18 years was almost 8.7 times higher for girls who seriously tried to lose or control their weight at age 18 (OR = 8.73, CI = 4.15–18.38, *p* < 0.001) and 14.5 times higher for overweight girls (OR = 14.46, CI = 1.84–113.75, *p* < 0.05). In contrast, the likelihood of wanting a thinner shape at 18 years was almost 10 times lower for underweight girls (according to self-reported BMI) at age 18 (OR = 0.10, CI = 0.03–0.33, *p* < 0.001). Self-esteem and having experienced sexual intercourse were not statistically significant.

**Table 2:** Factors measured at 18 years associated with BD at 18 years old among girls.

	Desire to be thinner at 18		
Variable	SE	OR	95% CI
Attempt to lose or control their weight	0.38	8.73^*^^*^^*^	4.15–18.38
Underweight (BMI)	0.59	0.10^*^^*^^*^	0.03–0.33
Overweight (BMI)	1.05	14.46^*^	1.84–113.75
Having experienced sexual intercourse	0.47	0.48	0.19–1.21
Self-esteem	0.04	0.93	0.87–1.00

Notes: *N* = 238. ^*^ *p* < 0.05; ^*^^*^^*^ *p* < 0.001.

### Factors associated with wanting a thinner or a bigger shape in boys at age 18

Multinomial logistic regressions were performed to predict the desire for a thinner or a bigger shape at age 18. Two risk factors at age 14 (BD and negative comments) predicted BD at age 18: BD and negative comments about weight (see Table 3). The likelihood of wanting a thinner shape at 18 years was almost 4.7 times higher for boys wanting a thinner shape at 14 years (OR = 4.68, CI = 1.57–14.02, *p* < 0.01), but 11.1 times lower for boys wanting a bigger shape (OR = 0.09, CI = 0.01–0.80, *p* < 0.05). The likelihood of wanting a thinner shape at age 18 was also 2.4 times higher for boys who received negative comments about their weight at age 14 (OR = 2.35, CI = 1.18–4.71, *p* < 0.05) compared with boys who did not report this behaviour. Self-esteem and control weight behaviours at age 14 did not predict wanting a thinner shape at age 18. No factors at age 14 predicted the desire for a bigger shape at age 18.

**Table 3:** Factors measured at 14 years old predicting BD at 18 years old among boys.

	Desire to be thinner at 18			Desire to be bigger at 18		
Variable	SE	OR	95% CI	SE	OR	95% CI
BD						
Desire to be thinner at 14	0.56	4.68^*^^*^	1.57–14.02	0.72	0.91	0.22–3.68
Desire to be bigger at 14	1.13	0.09^*^	0.01–0.80	0.54	2.78	0.97–7.95
Attempt to lose or control their weight	0.60	0.53	0.16–1.71	1.19	0.20	0.02–2.03
Receiving negative comments about their weight	0.35	2.35^*^	1.18–4.71	0.38	1.16	0.55–2.43
Self-esteem	0.05	1.02	0.93–1.12	0.05	0.99	0.90–1.10

Notes: *N* = 139. ^*^ *p* < 0.05; ^*^^*^ *p* < 0.01.

Two risk factors at age 18 were associated with the desire for a thinner or a bigger shape at age 18: low self-esteem and having experienced sexual intercourse (see Table 4). The likelihood of wanting a thinner shape or wanting a bigger shape at age 18 was associated with lower self-esteem at age 18 (OR = 0.84, CI = 0.75–0.95, *p* < 0.01; OR = .87, CI = 0.78–0.97, *p* < 0.05, respectively) and with having experienced sexual intercourse (OR = 3.36, CI = 1.15–9.84, *p* < 0.05; OR = 3.84, CI = 1.49–9.95, *p* < 0.01, respectively). The likelihood of wanting a thinner shape at 18 years was also almost 11.9 times higher among overweight boys (OR = 11.91, CI = 3.26–43.58, *p* < 0.001) and 6.3 times higher for boys who seriously tried to lose or control their weight at age 18 (OR = 6.29, CI = 1.49–26.57, *p* < 0.05). These factors were not associated with the desire for a bigger shape at age 18.

**Table 4:** Factors measured at 18 years old associated with BD at 18 years old among boys.

	Desire to be thinner			Desire to be bigger		
Variable	SE	OR	95% CI	SE	OR	95% CI
Attempt to lose or control their weight	0.74	6.29^*^	1.49–26.57	0.98	0.55	0.80–3.72
Overweight (BMI)	0.66	11.91^*^^*^^*^	3.26–43.58	1.16	0.32	0.03–3.10
Having experienced sexual intercourse	0.55	3.36^*^	1.15–9.84	0.49	3.84^*^^*^	1.49–9.95
Self-esteem	0.06	0.84^*^^*^	0.75–0.95	0.06	0.87^*^	0.78–0.97

Notes: *N* = 146. ^*^ *p* < 0.05; ^*^^*^ *p* < 0.01; ^*^^*^^*^ *p* < 0.001. Underweight (BMI) was not included because of the small sample size (*n* = 7).

## Discussion

Considering the high prevalence of BD and its negative consequences on mental health, this study aimed to examine the development of BD during adolescence as well as the factors associated with wanting a thinner or a bigger shape throughout this critical adolescent period. Although several longitudinal studies have been conducted on body image, no previous research had specifically investigated BD over time among girls and boys using a multidimensional framework.

BD appeared stable from ages 14 to 18 in boys and girls, which may seem contradictory to previous studies that reported an increase of BD over time (e.g. see Levine & Smolak, 2002). However, when we distinguished between the adolescents wanting a thinner shape and those wanting a bigger shape, we found BD to change over time, but only for girls. The proportion of adolescent girls wanting to be thinner increased with age, whereas the proportion of those wanting to be bigger decreased. Our results confirm the importance not only of studying BD, but also of looking at it in both directions, that is, the desire to be thinner and the desire to be bigger. In addition, it should be noted that a high proportion of girls who perceived their body as normal or even very thin still wanted a thinner shape. Meanwhile, the desire to be bigger was more common among boys than among girls. This finding likely relates to the cultural ideal of thinness for girls and muscles for boys (Seidah, Bouffard, & Vezeau, 2004). Previous research has also found that most girls with BD want a thinner shape, whereas some boys with BD want to be thinner and others want to be bigger (Presnell et al., 2004; Ricciardelli & McCabe, 2001a). We also observed that girls perceived themselves as bigger over time, but boys did not. This increase in shape in girls may be related to normal physical changes (such as weight gain) that occur during puberty (Richards, Boxer, Petersen, & Albrecht, 1990) and may be a factor in explaining the increase in girls wanting a thinner shape over time.

Consistent with one previous study (Presnell et al., 2004), we also found that boys tended to be dissatisfied with their shape when their BMI was below or above average, and girls tended to be dissatisfied when their BMI was average or above average (BD increasing linearly with BMI). Findings from this study are also in line with previous research indicating that underweight girls and average-weight boys are mostly satisfied with their own body image, whereas overweight adolescents of both genders are mostly dissatisfied with their body image (Cortese et al., 2010; Presnell et al., 2004; Richards et al., 1990). These results support the hypothesis that BD increases among girls as their bodies deviate from the ultrathin ideal (Graber, Brooks-Gunn, Paikoff, & Warren, 1994).

The finding that about half of the girls report dieting and using several strategies to control or lose weight (a substantially higher proportion than that found among adolescent boys) also concurs with previous studies (Eaton et al., 2008; McCabe & Ricciardelli, 2001; Neumark-Sztainer & Hannan, 2000; Neumark-Sztainer, Story, Hannan, Perry, & Irving, 2002; Neumark-Sztainer, Wall, Larson, Eisenberg, & Loth, 2011). As found in other studies (Eaton et al., 2008; Neumark-Sztainer et al., 2002, 2011), moderate weight control behaviours (e.g. reducing sugar and fat intake) were more common than extreme ones (e.g. skipping meals, using laxatives, purging). Nonetheless, weight control behaviours still raise public health concerns for adolescent girls with a healthy BMI who are dissatisfied with their body shape (Crow, Eisenberg, Story, & Neumark-Sztainer, 2006; Neumark-Sztainer et al., 2002).

Results of multivariate models showed that factors at age 14 predicting BD at age 18 are closely linked to physical appearance. BD at age 14 was the most significant factor in predicting BD at age 18 in both girls and boys (but only the desire for a thinner shape among boys). It appears that adolescents’ preoccupation with body image at an early age is maintained 4 years later. Another factor that predicted BD was weight control strategies for girls. As suggested by Bearman et al. (2006), self-reported dieting may also amplify feelings of BD, as it also predicts weight gain (Klesges, Isbell, & Klesges, 1992). The number of negative comments received from others about weight control practices also predicted the desire for a thinner shape for both girls and boys. This result is similar to other studies that found a relationship between BD and weight teasing or conversations with friends regarding appearance (Lawler & Nixon, 2011; Paxton et al., 2006a). Adolescents’ surroundings thus appear to be an important factor related to BD. Finally, no factors predicted the desire for a bigger shape among boys, possibly because the sample of boys wanting a bigger shape was small. Furthermore, it was not possible to study the desire for a bigger shape among girls, as only 12 girls had this desire. Further studies with larger samples are thus needed to investigate factors related to the desire for a bigger shape among adolescent boys and girls.

Results of multivariate models of factors at age 18 associated with BD at age 18 reveal that BMI was the strongest factor associated with the desire for a thinner shape at age 18 for both boys and girls. Our results are also consistent with other studies that demonstrated an association between BMI and body image dissatisfaction (Cantin & Stan, 2010; Cortese et al., 2010; Ohring, Grabe, & Brooks-Gunn, 2002; Presnell et al., 2004). Interestingly, although BMI was marginally significant (*p* = 0.06), it was not related to the desire for a bigger shape among boys. Weight control behaviours at age 18 were also related to the desire for a thinner shape among both girls and boys. Although we found that weight control behaviours at age 14 predicted BD among girls at age 18, it may also be a consequence of BD. BD may lead to the development of weight control behaviours, to meet the norms of beauty, and these weight control behaviours may in turn increase BD.

To date, the association between body image and having experienced sexual intercourse has not been widely studied among adolescents. One study found no association between weight concerns and having experienced sexual intercourse in adolescent girls (Halpern, Udry, Campbell, & Suchindran, 1999). Another study found an association between negative body image and having experienced sexual intercourse in adolescent girls and boys, but it remained significant only for girls after controlling for individual factors (Valle, Røysamb, Sundby, & Klepp, 2009). We found an association between BD (for the desire for a thinner and a bigger shape) and having experienced sexual intercourse at age 18 in boys. It may be that having experienced sexual intercourse increases awareness of one's physical appearance. Boys may want to change their body (have a bigger or a thinner body) to fit norms of beauty in order to increase physical attractiveness. Sexual intercourse could also be a consequence of BD: adolescent boys dissatisfied with their body may engage in sexual activities to increase their self-confidence. Future studies should explore how adolescent sexuality (such as the circumstances of the first intercourse and sexual risk taking) could be associated with BD.

Self-esteem was found to be related to BD in previous longitudinal studies (e.g. Cantin & Stan, 2010; Paxton et al., 2006a), with one exception (Tiggeman, 2005). Tiggeman's (2005) results suggest that body image dissatisfaction predicts low self-esteem, but not the reverse, which suggests that there would be no temporal precedence for low self-esteem in BD. In our study, only low self-esteem at age 18 was associated with BD among boys, which might suggest that it could be more a consequence of BD. Further longitudinal studies investigating self-esteem and BD over a longer period with several time measurements are needed to better understand their interrelationships. Nonetheless, it seems that self-esteem is less associated with BD compared with factors associated with physical appearance, such as health control strategies and BMI. It may also be that self-esteem has more impact on body image dimensions other than BD, which should be further studied.

### Strengths and limitations

This study used a longitudinal design to describe the development of BD and identify associated factors over a 4-year period marked by important changes in adolescence. Other strengths include the use of a community-based sample, the comparison of adolescent boys and girls, and the assessment of different predictors and associated factors that could be related to BD. Several limitations are important to mention: as in many other studies, our measures of weight control, negative comments and sexual intercourse were based on a single-item categorical response, which may be imprecise. Future studies should use more comprehensive measures with validated scales to capture each of these constructs in much greater detail. Attrition of our longitudinal sample could limit the generalisation of the results. Finally, BMI was based on self-reported heights and weights at 18 years of age; self-reported BMI is considered valid (Sherry, Jefferds, & Grummer-Strawn, 2007).

## Conclusions

In summary, our results provide new insights on BD development because our study was the first to investigate BD prospectively with a figural drawing scale. Overall, the data suggest that adolescent girls report higher levels of BD compared with boys throughout adolescence and that it is highly predicted by previous BD. Moreover, concerns about one's weight (weight control behaviours) and those stemming from suggestions by others (negative comments) at age 14 were associated with negative consequences, such as increased BD, 4 years later at age 18. Finally, our results also confirm the high proportion of girls concerned with body image. More specifically, adolescent girls are more likely to strive towards having a thinner shape and to have tried to lose or control their weight using harmful control strategies. These results confirm the importance of assessing not only being satisfied or not with current shape, but also specific aspects of BD, such as the desire to be thinner or to be bigger. It is possible that girls are more influenced than boys by the media, as other studies have found (Polce-Lynch, Myers, Kliewer, & Kilmartin, 2001), or that norms of beauty are more targeted towards girls. In the future, it could be interesting to study whether changes in the media could affect BD among adolescent boys and girls. Nonetheless, our societal approach to body image is clearly problematic. Intervention programmes should focus on helping adolescents accept their body and concomitantly teach them about healthy dietary attitudes and behaviours.

## Societal preventive implications

Preventive programmes should target a broad spectrum of weight-related problematic conditions and behaviours, such as obesity, eating disorders and BD (Neumark-Sztainer, 2005; Neumark-Sztainer et al., 2006a). These programmes should optimally start during childhood because BD is already high at age 14, according to our findings, and also during childhood (Clark & Tiggemann, 2007; Deleel, Hughes, Miller, Hipwell, & Theodore, 2009; Mendelson et al., 1996; Ricciardelli & McCabe, 2001b). These preventive programmes should also target the negative impacts of dieting and comments about weight, which are important risk factors of BD. Governments should promote more initiatives to reduce BD, such as the Quebec Charter for a Healthy and Diverse Body Image (Quebec Government, 2011). Finally, these preventive programmes should aim to reduce the high prevalence of BD, obesity and eating disorders among adolescents in many industrial societies (Neumark-Sztainer et al., 2002).

## References

[cit0001] Almeida S., Severo M., Araújo J., Lopes C., Ramos E. (2012). Body image and depressive symptoms in 13-year-old adolescents. Journal of Paediatrics and Child Health.

[cit0002] Bearman S. K., Presnell K., Martinez E., Stice E. (2006). The skinny on body dissatisfaction: A longitudinal study of adolescent girls and boys. Journal of Youth and Adolescence.

[cit0003] Bucchianeri M. M., Arikian A. J., Hannan P. J., Eisenberg M. E., Neumark-Sztainer D. (2013). Body dissatisfaction from adolescence to young adulthood: Findings from a 10-year longitudinal study. Body Image.

[cit0004] Calzo J. P., Sonneville K. R., Haines J., Blood E. A., Field A. E., Austin S. B. (2012). The development of associations among body mass index, body dissatisfaction, and weight and shape concern in adolescent boys and girls. Journal of Adolescent Health.

[cit0005] Cantin S., Stan S. N. (2010). Les relations avec les pairs à l'adolescence comme facteurs de risque de l'insatisfaction à l'égard de l'image corporelle [Peer relations in adolescence as risk factors for body image dissatisfaction]. Canadian Journal of Behavioural Science.

[cit0006] Cash T. F., Pruzinsky T. (2011). Body image: A Handbook of theory, research, and clinical Practice.

[cit0007] Chen L. -J., Fox K. R., Haase A. M., Ku P. -W. (2010). Correlates of body dissatisfaction among Taiwanese adolescents. Asia Pacific Journal of Clinical Nutrition.

[cit0008] Clark L., Tiggemann M. (2007). Sociocultural influences and body image in 9- to 12-year-old girls: The role of appearance schemas. Journal of Clinical Child and Adolescent Psychology.

[cit0009] Cortese S., Falissard B., Pigaiani Y., Banzato C., Bogoni G., Pellegrino M., Maffeis C. (2010). The relationship between body mass index and body size dissatisfaction in young adolescents: Spline function analysis. Journal of the American Dietetic Association.

[cit0010] Crespo C., Kielpikowski M., Jose P. E., Pryor J. (2010). Relationships between family connectedness and body satisfaction: A longitudinal study of adolescent girls and boys. Journal of Youth and Adolescence.

[cit0011] Crow S., Eisenberg M. E., Story M., Neumark-Sztainer D. (2006). Psychosocial and behavioral correlates of dieting among overweight and non-overweight adolescents. Journal of Adolescent Health.

[cit0012] Crow S., Eisenberg M. E., Story M., Neumark-Sztainer D. (2008). Suicidal behavior in adolescents: Relationship to weight status, weight control behaviors and body dissatisfaction. International Journal of Eating Disorders.

[cit0013] Deleel M. L., Hughes T. L., Miller J. A., Hipwell A., Theodore L. A. (2009). Prevalence of eating disturbance and body image dissatisfaction in young girls: An examination of the variance across racial and socioeconomic groups. Psychology in the Schools.

[cit0014] Eaton D. K., Kann L., Kinchen S., Shanklin S., Ross J., Hawkins J., Wechsler H. (2008). Youth risk behavior surveillance – United States. MMWR. Surveillance Summaries.

[cit0015] Eisenberg M. E., Neumark-Sztainer D., Paxton S. J. (2006). Five-year change in body satisfaction among adolescents. Journal of Psychosomatic Research.

[cit0016] Gardner R. M., Brown D. L. (2010). Body image assessment: A review of figural drawing scales. Personality and Individual Differences.

[cit0017] Graber J. A., Brooks-Gunn J., Paikoff R. L., Warren M. P. (1994). Prediction of eating problems: An 8-year study of adolescent girls. Developmental Psychology.

[cit0018] Halpern C. T., Udry J. R., Campbell B., Suchindran C. (1999). Effects of body fat on weight concerns, dating, and sexual activity: A longitudinal analysis of black and white adolescent girls. Developmental Psychology.

[cit0019] Health Canada (2003). Canadian guidelines for body weight classification in adult.

[cit0020] Holsen I., Kraft P., Roysamb E. (2001). The relationship between body image and depressed mood in adolescence: A 5-year longitudinal panel study. Journal of Health Psychology.

[cit0021] Jones L. R., Fries E., Danish S. J. (2007). Gender and ethnic differences in body image and opposite sex figure preferences of rural adolescents. Body Image.

[cit0022] Klesges R. C., Isbell T. R., Klesges L. M. (1992). Relationship between dietary restraint, energy intake, physical activity, and body weight: A prospective analysis. Journal of Abnormal Psychology.

[cit0023] Lawler M., Nixon E. (2011). Body dissatisfaction among adolescent boys and girls: The effects of body mass, peer appearance culture and internalization of appearance ideals. Journal of Youth and Adolescent.

[cit0024] Levine M. P., Smolak L., Cash T. F., Pruzinsky T. (2002). Body image development in adolescence.

[cit0025] McCabe M. P., Ricciardelli L. A. (2001). Parent, peer, and media influences on body image and strategies to both increase and decrease body size among adolescent boys and girls. Adolescence.

[cit0027] Mendelson B. K., White D. R., Mendelson M. J. (1996). Self-esteem and body esteem: Effects of gender, age, and weight. Journal of Applied Developmental Psychology.

[cit0026] Menzel J. E., Krawczyk R., Thompson J. K., Cash T. F., Smolak L. (2011). Attitudinal assessment of body image for adolescents and adults.

[cit0028] Neumark-Sztainer D. (2005). Can we simultaneously work toward the prevention of obesity and eating disorders in children and adolescents?. International Journal of Eating Disorders.

[cit0029] Neumark-Sztainer D., Hannan P. J. (2000). Weight-related behaviors among adolescent girls and boys: Results from a national survey. Archives Pediatrics and Adolescent Medecine.

[cit0030] Neumark-Sztainer D., Levine M. P., Paxton S. J., Smolak L., Piran N., Wertheim E. H. (2006a). Prevention of body dissatisfaction and disordered eating: What next?. Eating disorders.

[cit0031] Neumark-Sztainer D., Paxton S. J., Hannan P. J., Haines J., Story M. (2006b). Does body satisfaction matter? Five-year longitudinal associations between body satisfaction and health behaviors in adolescent females and males. Journal of Adolescent Health.

[cit0032] Neumark-Sztainer D., Story M., Hannan P. J., Perry C. L., Irving L. M. (2002). Weight-related concerns and behaviors among overweight and nonoverweight adolescents. Archives of Pediatrics and Adolescent Medicine.

[cit0033] Neumark-Sztainer D., Wall M., Larson I. M., Eisenberg M. E., Loth K. (2011). Dieting and disordered eating behaviors from adolescence to young adulthood: Findings from a 10-year longitudinal study. Journal of the American Dietetic Association.

[cit0034] Ohring R., Graber J. A., Brooks-Gunn J. (2002). Girls’ recurrent and concurrent body dissatisfaction: Correlates and consequences over 8 years. International of Journal of Eating Disorders.

[cit0035] Paxton S. J., Eisenberg M. E., Neumark-Sztainer D. (2006a). Prospective predictors of body dissatisfaction in adolescent girls and boys: A five-year longitudinal study. Developmental Psychology.

[cit0036] Paxton S. J., Neumark-Sztainer D., Hannan P. J., Eisenberg M. E. (2006b). Body dissatisfaction prospectively predicts depressive mood and low self-esteem in adolescent girls and boys. Journal of Clinical Child and Adolescent Psychology.

[cit0037] Petroski E. L., Pelegrini A., Glaner M. F. (2012). Reasons and prevalence of body image dissatisfaction in adolescents. Cien Saude Colet.

[cit0038] Polce-Lynch M., Myers B. J., Kliewer W., Kilmartin C. (2001). Adolescent self esteem and gender: Exploring relations to sexual harassment, body image, media influence, and emotional expression. Journal of Youth and Adolescent.

[cit0039] Presnell K., Bearman S. K., Stice E. (2004). Risk factors for body dissatisfaction in adolescent boys and girls: A prospective study. International Journal of Eating Disorders.

[cit0040] Quebec Government The Quebec charter for a healthy and diverse body image. http://www.ijoinonline.com.

[cit0041] Ricciardelli L. A., McCabe M. P. (2001a). Dietary restraint and negative affect as mediators of body dissatisfaction and bulimic behavior in adolescent girls and boys. Behaviour Research and Therapy.

[cit0042] Ricciardelli L. A., McCabe M. P. (2001b). Children's body image concerns and eating disturbance: A review of the literature. Clinical Psychology Review.

[cit0043] Richards M. H., Boxer A. W., Petersen A. C., Albrecht R. (1990). Relation of weight to body image in pubertal girls and boys from two communities. Developmental Psychology.

[cit0044] Rosenberg M. (1965). Rosenberg's self-esteem scale.

[cit0045] Seidah A., Bouffard T., Vezeau C. (2004). Perceptions de soi à l'adolescence: Différences entre filles et garçons [Self-perceptions in adolescence: Differences between girls and boys]. Enfance.

[cit0046] Sherry B., Jefferds M. E., Grummer-Strawn L. M. (2007). Accuracy of adolescent self-report of height and weight in assessing overweight status: A literature review. Archives of Pediatrics and Adolescent Medicine.

[cit0047] Stice E. (2002). Risk and maintenance factors for eating pathology: A meta-analytic review. Psychological Bulletin.

[cit0048] Stice E., Bearman S. K. (2001). Body-image and eating disturbances prospectively predict increases in depressive symptoms in adolescent girls: A growth curve analysis. Developmental Psychology.

[cit0049] Stice E., Marti C. N., Durant S. (2011). Risk factors for onset of eating disorders: Evidence of multiple risk pathways from an 8-year prospective study. Behaviour Research Therapy.

[cit0050] Stice E., Whitenton K. (2002). Risk factors for body dissatisfaction in adolescent girls: A longitudinal investigation. Developmental Psychology.

[cit0051] Thompson M. A. (1993). Fat distribution, site-specific body dissatisfaction, and perceived difficulty in regional weight loss as risk factors for disordered eating behavior and body image disturbances.

[cit0052] Thompson M. A., Gray J. J. (1995). Development and validation of a new body-image assessment scale. Journal of Personality Assessment.

[cit0053] Thompson J. K., Heinberg L. J., Altabe M. N., Tantleff-Dunn S. (1999). Exacting beauty: Theory, assessment, and treatment of body image disturbance.

[cit0054] Tiggemann M. (2005). Body dissatisfaction and adolescent self-esteem: Prospective findings. Body Image.

[cit0055] Tomkins C. (2006). An introduction to non-parametric statistics for health scientists. University of Alberta Health Sciences Journal.

[cit0056] Valle A. K., Røysamb E., Sundby J., Klepp K. I. (2009). Parental social position, body image, and other psychosocial determinants and first sexual intercourse among 15- and 16-year olds. Adolescence.

[cit0057] Vallières E. F., Vallerand R. J. (1990). Traduction et validation canadienne-française de l'échelle de l'estime de soi de Rosenberg [French-Canadian translation and validation of Rosenberg's Self-Esteem Scale]. International Journal of Psychology.

[cit0058] Wertheim E. H., Paxton S. J., Cash T. F., Pruzinsky T. (2011). Body image development in adolescent girls.

[cit0059] Wertheim E. H., Paxton S. J., Tilgner L. (2004). Test-retest reliability and construct validity of contour drawing rating scale scores in a sample of early adolescent girls. Body Image.

[cit0060] Wilkosz M. E., Chen J. L., Kenndey C., Rankin S. (2011). Body dissatisfaction in California adolescents. Journal of the American Academy of Nurse Practitioners.

[cit0061] Woelders L. C. S., Larsen J. K., Scholte R. H. J., Cillessen A. H. N., Engels R. C. M. E. (2010). Friendship group influences on body dissatisfaction and dieting among adolescent girls: A prospective study. Journal of Adolescent Health.

